# Global analysis of gene expression profiles in physic nut (*Jatropha curcas* L.) seedlings exposed to drought stress

**DOI:** 10.1186/s12870-014-0397-x

**Published:** 2015-01-21

**Authors:** Chao Zhang, Lin Zhang, Sheng Zhang, Shuang Zhu, Pingzhi Wu, Yaping Chen, Meiru Li, Huawu Jiang, Guojiang Wu

**Affiliations:** Key Laboratory of Plant Resources Conservation and Sustainable Utilization, South China Botanical Garden, Chinese Academy of Sciences, Guangzhou, 510650 China; University of Chinese Academy of Sciences, Beijing, 100049 China; Department of Environmental Engineering and Chemistry, Luoyang Institute of Science and Technology, Luoyang, 471023 China; Department of Biology, South University of Science and Technology of China, Shenzhen, 518055 China

**Keywords:** Physic nut (*Jatropha curcas* L.), drought stress, gene expression profiles, abscisic acid, waxes and fatty acids, endoplasmic reticulum stress response, senescence

## Abstract

**Background:**

Physic nut (*Jatropha curcas* L.) is a small perennial tree or large shrub, which is well-adapted to semi-arid regions and is considered to have potential as a crop for biofuel production. It is now regarded as an excellent model for studying biofuel plants. However, our knowledge about the molecular responses of this species to drought stress is currently limited.

**Results:**

In this study, genome-wide transcriptional profiles of roots and leaves of 8-week old physic nut seedlings were analyzed 1, 4 and 7 days after withholding irrigation. We observed a total of 1533 and 2900 differentially expressed genes (DEGs) in roots and leaves, respectively. Gene Ontology analysis showed that the biological processes enriched in droughted plants relative to unstressed plants were related to biosynthesis, transport, nucleobase-containing compounds, and cellular protein modification. The genes found to be up-regulated in roots were related to abscisic acid (ABA) synthesis and ABA signal transduction, and to the synthesis of raffinose. Genes related to ABA signal transduction, and to trehalose and raffinose synthesis, were up-regulated in leaves. Endoplasmic reticulum (ER) stress response genes were significantly up-regulated in leaves under drought stress, while a number of genes related to wax biosynthesis were also up-regulated in leaves. Genes related to unsaturated fatty acid biosynthesis were down-regulated and polyunsaturated fatty acids were significantly reduced in leaves 7 days after withholding irrigation. As drought stress increased, genes related to ethylene synthesis, ethylene signal transduction and chlorophyll degradation were up-regulated, and the chlorophyll content of leaves was significantly reduced by 7 days after withholding irrigation.

**Conclusions:**

This study provides us with new insights to increase our understanding of the response mechanisms deployed by physic nut seedlings under drought stress. The genes and pathways identified in this study also provide much information of potential value for germplasm improvement and breeding for drought resistance.

**Electronic supplementary material:**

The online version of this article (doi:10.1186/s12870-014-0397-x) contains supplementary material, which is available to authorized users.

## Background

Drought stress is one of the most important limitations to plant growth and crop yield [1]. There are two major strategies by which plants resist drought stress: drought avoidance and drought tolerance [2]. Drought avoidance includes a number of protective mechanisms that delay or prevent the negative impact of drought on plants, while drought tolerance is the potential of plants to adapt to stress conditions [3]. Plant responses to drought stress can result in alterations to the structures of membranes, cell walls and whole organs, as well as accumulation of compatible solutes to act as osmoprotectants, changes in cellular redox balance, and the synthesis of detoxifying enzymes and transporters [3,4].

Plant hormones and other signals mediate the changes in plant structure and metabolic pathways that occur under drought stress. Previous studies of genes involved in drought responses and mutations in these genes have identified important signaling substances and signal transduction pathways in plants; the latter are divided into abscisic acid (ABA)-dependent and ABA-independent signaling pathways [5,6]. ABA can induce the expression of stress-related genes, promote stomatal closure and induce the accumulation of many osmotic stress-induced proteinogenic amino acids [3,6]. The concentration of ABA in plants is dependent on the rates of its biosynthesis and catabolism. NCED (9-*cis*-epoxycarotenoid dioxygenase) catalyzes the key step in the ABA biosynthesis pathway [7], while CYP707A3 (a cytochrome P450 protein which has ABA 8'-hydroxylase activity) [8] and ABA GTase (ABA glucosyltransferase) [9] play roles in ABA catabolism. Immediately following its biosynthesis, ABA acts via a signaling pathway with participants that include receptors of the PYR/PYL family, protein phosphatase 2C (PP2C), Serine/threonine-protein Kinase SRK (SnRK), and ABA Responsive Element Binding Factors (AREBs/ABFs) [10,11]. Downstream of this signaling pathway, the expression of partially responsive to desiccation (RD) genes, *RD*22, *RD*26, *RD*20A, and *RD*29B, is induced in response to drought in an ABA-dependent manner [6,12]. A number of drought stress induced genes, such as *RD29A* and *ERD1*, are ABA-independent [13], and are regulated by the ABA-independent transcription factors DREB2A [14] and members of the NAC and HD-ZIP families of proteins [6,15].

In addition to ABA signaling, there are other signaling pathways involved in drought stress, such as ethylene (ETH) signaling and endoplasmic reticulum (ER) stress response signaling. ETH is an important gaseous plant hormone, which has a wide range of functions in the regulation of plant growth and senescence [16-18]. ETH is synthesized from the substrate L-methionine in many tissues, and the rate-limiting enzymes in the biosynthetic pathway are 1-Aminocyclopropane-1-Carboxylate Synthase (ACS) and Aminocyclopropane Carboxylate Oxidase (ACO) [19,20]. After synthesis, ETH acts via Ethylene-Responsive Transcription Factors (ERFs) [21,22] and ETH receptors; *Arabidopsis* has five ETH receptors, ethylene response 1 (ETR1), ETR2, ethylene response sensor 1 (ERS1), ERS2, and ethylene insensitive 4 (EIN4) [23]. The ER stress response is activated by unfolded proteins that accumulate in the ER when plants are exposed to adverse environments [24]. In plants, there are two signal transduction pathways that can response to ER stress; one is mediated by membrane-associated transcription factors (bZIP17 and bZIP28); the other is dependent on a dual protein kinase, RNA-splicing factor IRE1 (inositol-requiring enzyme 1), which splices the mRNA encoding bZIP60 [24-26]. Under mild or short-term drought, signaling from IRE1 activates autophagy, a cell-sparing process, but under severe drought, ER stress leads to cell death [24].

Drought stress induces a large range of physiological and biochemical responses in plants, such as osmoprotectant synthesis, wax biosynthesis and changes in fatty acid composition. The biosynthesis of osmoprotectants is particularly important for plant resistance to drought stress, and osmoprotectants can include amino acid, amines and carbohydrates. The most common osmoprotectants are proline (Pro), γ-aminobutyric acid (GABA), glycine betaine (GB), fructans, starch, mono- and disaccharides, trehalose (Tre), and raffinose family oligosaccharides (RFO) [3]. Wax, the thin hydrophobic layer laid down on the leaf surface in many species, can protect plants from nonstomatal water loss under drought conditions [27,28]. In *Arabidopsis*, wax is synthesized via a pathway terminating in the enzyme wax synthase/diacylglycerol acyltransferase (WSD); *WSD* is regulated by MYB96 and *CER*, especially under drought conditions [29,30]. Previous studies have shown that fatty acid composition may be changed when plants are exposed to drought stress, and higher unsaturated fatty acid contents may increase; this is believed to maintain the fluidity and stability of cellular membranes [31,32].

Global transcriptomic data obtained by high-throughput sequencing have provided new insights to increase our understanding of complex stress response mechanisms, identify key metabolic pathway genes as targets for genetic engineering to improve stress tolerance, and discover novel stress response pathways [9]. For instance, gene expression profiling of drought responsiveness in rice revealed temporal and spatial regulation mediated through various developmental cues and environmental stimuli [33]. Genome wide expression profiling of two accessions of *Gossypium herbaceum* revealed the molecular mechanism underlying the physiological response of the better-adapted accession under drought stress [34].

Physic nut (*Jatropha curcas* L.) is a small perennial tree or large shrub, which belongs to the family Euphorbiaceae. It is well-adapted to semi-arid regions and considered to have potential as a renewable biofuel plant. Previous studies have shown that physic nut plants can maintain a high rate of growth and biomass increase even under a water-deficit of 40% Plant Available Water [35,36]. Other reports have demonstrated that physic nut plants can resist drought stress by accumulating osmoprotectants [37,38], reducing stomatal conductance and the biomass of aerial parts [39] and scavenging Reactive Oxygen Species (ROS) [40-42]. Under severe drought stress, physic nut plants show drought avoidance behavior, with a typical water saving strategy characterized by strict stomatal regulation and leaf drop [43,44]. Recently, several genes have been cloned and transformed into physic nut to improve its drought tolerance; they include those encoding D-myo-inositol-3-phosphate synthase (*JcMIPS*) [45], phosphopantetheine adenylyltransferase (*AtPPAT*) and the B subunit of the nuclear factor Y (*AtNF-YB*) [46]. Despite these studies, there have been few reports on the specific genes and mechanisms that participate in the response to drought stress in physic nut, especially with respect to spatiotemporal patterns of expression. In the present study, we analyzed gene expression profiles of physic nut roots and leaves 1, 4 and 7 days after withholding irrigation. By carrying out annotation of the functions of the genes identified, we found that the pathways that changed significantly in physic nut plants under drought stresses were related to ABA, ethylene and ER signaling. The contents of proline and chlorophyll, the composition of fatty acids in leaves and the genes involved in these pathways were analyzed.

## Methods

### Plant materials and experimental treatment

Physic nut (*J. curcas*) cultivar GZQX0401 was used in this study; it was introduced from Guizhou province and domesticated in the South China Botanic Garden, China Academy of Science. The seeds were germinated in substrates of sand and soil (3:1) in a greenhouse illuminated with natural sunlight (day/night ≈ 14 h/10 h; daily temperature 25 ~ 30°C). For drought treatment, six-leaf seedlings of physic nut planted in pots were used [47]. The group that was irrigated daily with Hoagland nutrient solution [48] was treated as the control, while the group from which irrigation was withheld represented the drought stress treatment. Based on observed changes in net photosynthesis rate (Pn) in physic nut leaves under drought stress, irrigated and unirrigated seedlings were sampled at three time points. They were: an early point (1 day after withholding irrigation, 1 DAWI); the point at which rapid reduction in Pn was initiated (4 DAWI; Pn, transpiration rate and stomatal conductance had decreased to ca. 80% of those in the control), and the point at which net photosynthesis rate had reached a very low level (7 DAWI; Pn, transpiration rate, and stomatal conductance had decreased to less than 20% of the control). The soil relative water content was 10.12% ± 1.10% at 7 DAWI. Root samples comprised all root tips ca. 5–10 mm long, while leaf samples consisted of blades of the third fully expanded leaf from the apex. Samples were harvested from three seedlings for each time point, and the collection was repeated three times to obtain physiological data. Two replicates used for sequencing were prepared in the same month in two years and samples were frozen immediately in liquid nitrogen and stored at −80°C.

### RNA isolation, digital gene expression library preparation and sequencing

Total RNA was extracted from root or leaf samples using the CTAB method [49]. The isolated RNA was subsequently treated with RNase-Free DNase I (Roche, http://www.roche.com).

Two biological replicates from leaves and roots sampled at each of the three time points, from both drought-stressed and control plants, were sequenced, making a total of 24 sequencing samples. Tag libraries from the RNA samples were prepared in parallel using an Illumina gene expression sample preparation kit and sequenced using the Illumina GAII platform at BGI-Shenzhen (http://en.genomics.cn/navigation/index.action) [50]. For gene expression analysis, the number of expressed tags was calculated and then normalized to TPM (number of transcripts per million tags) [51]. The sequencing saturation statistics are shown in Additional file 1: Table S1.

### Availability of supporting data

Information about the genomic sequences and predicted protein-encoding genes is available at DDBJ/EMBL/GenBank under the accession AFEW00000000. The version described in this paper is the first version, AFEW01000000. The raw data of gene expression profiles were submitted to the sequence read archive (SRA) at NCBI (accession number PRJNA257901).

### Identification of differentially expressed genes and Gene Ontology analysis

Differentially expressed genes (DEGs) were identified by IDEG6 (http://telethon.bio.unipd.it/bioinfo/IDEG6_form/index.html) using the test of Audic and Claverie, with a significance threshold of 0.01 and Bonferroni Correction [52]. Only those genes whose expression levels changed more than two-fold between control and drought conditions were treated as being up- or down-regulated. To further confirm that DEGs had been identified accurately, the TPMs of drought stress (up-regulated genes) or control conditions (down-regulated genes) were limited to be more than threshold values that were defined as the 20% values of the average of TPM of all expressed genes [47]. All the genes were annotated by Blastp against the NCBI non-redundant protein sequences (nr) database and the *Arabidopsis* Information Resource (TAIR) Proteins database. The annotated genes were then analyzed for Gene Ontology (GO) function using AgBase GORetriever and GOSlimViewer (http://agbase.msstate.edu/index.html) [53].

### Verification of gene expression profile results by quantitative real-time PCR

To validate the veracity of the digital expression data, genes involved in ABA biosynthesis and signal transduction were tested by quantitative real-time PCR (qRT-PCR). RNA was extracted as described in the RNA isolation section. First-strand cDNA was synthesized from 2 μg total RNA using the M-MLV reverse transcriptase (Promega, http://www.promega.com). *JcActin*, a housekeeping gene, was used as the internal control. Primers were designed using Primer Premier 6 (http://www.premierbiosoft.com/primerdesign/index.html); the primer sequences used were 5’ taatggtccctctggatgtg 3’ (Forward primer, F) and 5’ agaaaagaaaagaaaaaagcagc (Reverse primer, R) for *JcActin* [47,49,54], 5’ gggcattctggaattgctaggctat 3’ (F) and 5’ cacaaggaagaacacggacatggt 3’ (R) for JC_C 100001845, 5’ tggtgatcggatcttgcatgactc 3’ (F) and 5’ tgactctttcttcctaagcggttcc 3’ (R) for JC_C 100015061, 5’ tacagcagcagcagcagcag 3’ (F) and 5’ ccacacctcctaatccaaccattcc 3’ (R) for JC_C 100011364, 5’ gccaccaattcagccaaaccaatg 3’ (F) and 5’ gcccactaggaaggagttcagatac 3’ (R) for JC_C100019357. qRT-PCR was performed with a Light Cycler 480II Real-Time PCR System (Roche, http://www.roche.com) using the SYBR green PCR kit (TaKaRa Code: DRR041A) with three technical replicates. The ΔΔC_T_ method of relative gene quantification was used to calculate the expression level of each gene in the two tissues at the three stages of drought stress. Three biological replicates were used for the qRT-PCR analysis.

### Chemical substance assays

Proline was extracted from leaves at 7 DAWI with 3% sulfosalicylic acid, and then reacted with acid ninhydrin reagent. After extracting with methylbenzene, absorbances at 520 nm were measured [55]. For the determination of raffinose, stachyose and trehalose, 4 g of leaves and 1 g of roots were ground with liquid nitrogen and extracted with ultrapure water for 30 min at 80°C. After centrifugation at 5000 g, the supernatant was passed through 0.2 μm filters. Then absolute ethanol was added to the filtrate to make the ethanol content up to 80%, and the mixture was left overnight at 4°C. Subsequently, the centrifugation and filtration steps were repeated. The supernatant was dried using a rotary evaporator (EYEL4, Japan), and dissolved in 400 μl ultrapure water. The oligosaccharide contents were analyzed by high performance liquid chromatography (HPLC, Waters e2695, USA) with a NH_2_ column (Waters, 250 mm × 4.6 mm, 3.5 μm) and detected by evaporative light scattering detector (ELSD, Alltech 3300, USA) [56,57]. Chlorophylls were extracted from leaves at 7 DAWI with 80% acetone overnight. The absorbances at 645 nm and 663 nm were measured, and the chlorophyll contents were calculated as described by Arnon [58]. The fatty acid composition of leaves at 7 DAWI was analyzed by methyl esterification and gas chromatography [59,60]. All experiments included three biological repeats, and the data were analyzed with a Duncan test [61] using the SAS software package (http://www.sas.com/en_us/software/sas9.html).

## Results

### General features of drought stress responsive genes

**Figure 1:**
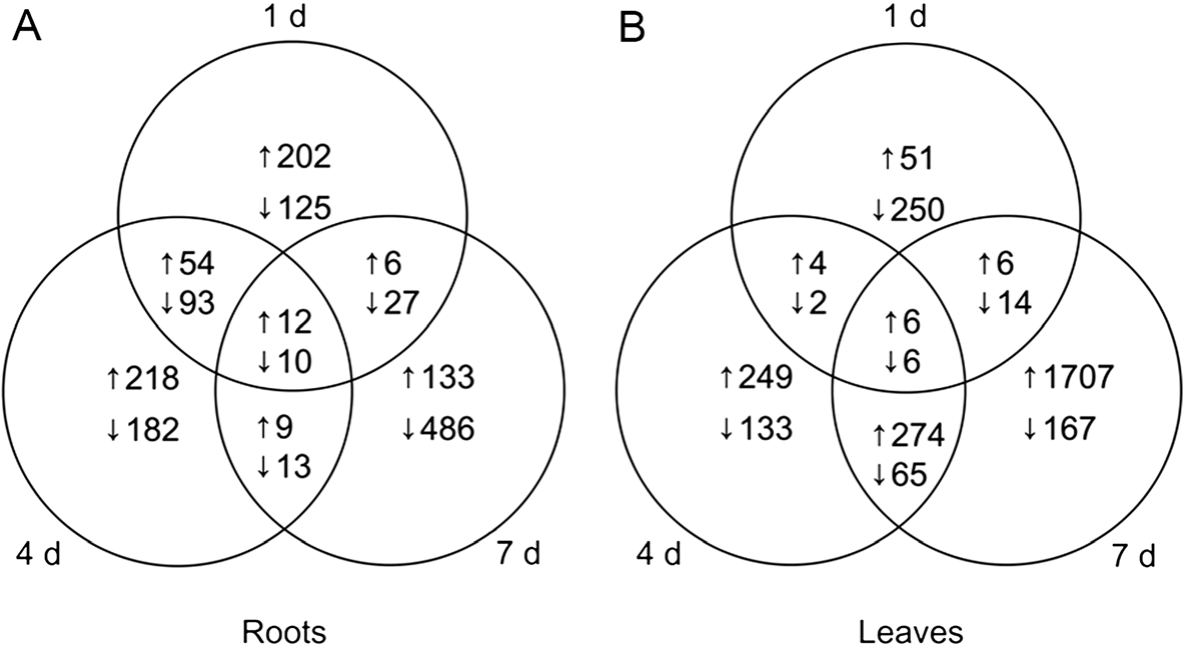
**Venn diagram of DEGs up- and down-regulated 1, 4 and 7 days after withholding irrigation. A.** Venn diagram of DEGs up- and down-regulated in roots. **B.** Venn diagram of DEGs up- and down-regulated in leaves. “↑” and “↓” are standing for up- and down-regulated DEGs, respectively.

In identifying differentially expressed genes (DEGs), for a gene to be considered differentially expressed, the gene expression ratio had to exceed two for each of the replicates. In addition, its expression level at a given time point had to be at least 20% higher than the average level at that time point, either in stressed samples (up-regulated genes) or in control samples (down-regulated genes). The total numbers of genes that met these criteria were 1533 and 2900 in roots and leaves respectively. The total numbers of DEGs at 1 d, 4 d and 7 d were 846, 1250 and 2849 respectively (Figure 1). Genes that showed up- or down-regulation at two or three time points in roots and/or leaves under drought treatment are listed in Additional file 2: Table S2.

**Figure 2:**
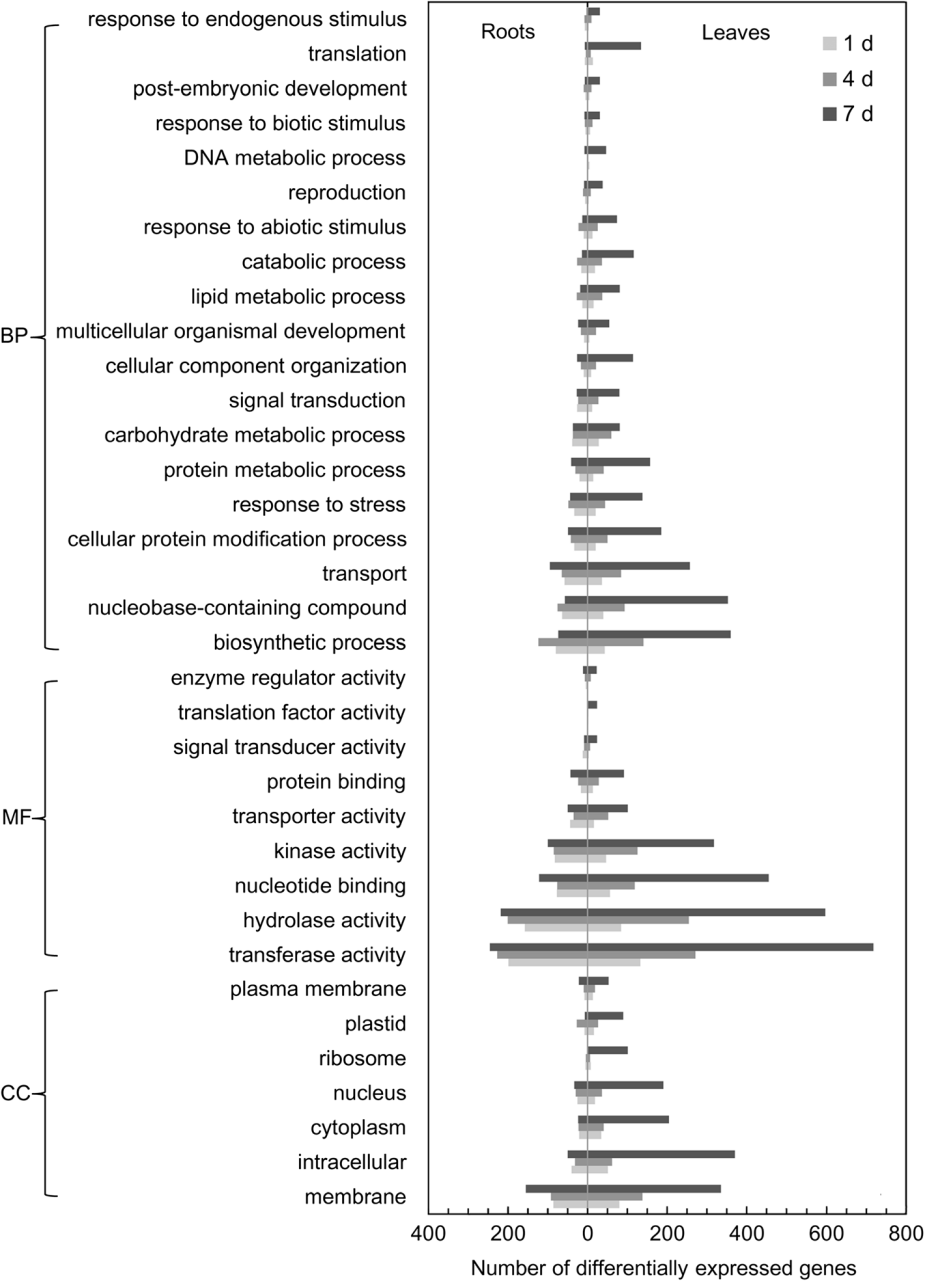
**Gene Ontology (GO) analysis.** BP, Biological Process; MF, Molecular Function; CC, Cellular Component.

All the DEGs were annotated using the results of blastp queries against the NCBI non-redundant protein sequences database and the *Arabidopsis* Information Resource Proteins database and the results were analyzed in AgBase (http://www.agbase.msstate.edu/). These GO annotations, including those for Cellular Component, Molecular Function and Biological Process, were collected and used to construct graphs (Figure 2). The three most highly enriched GO terms for Cellular Component were membrane, intracellular and cytoplasm, while transferase activity, hydrolase activity and nucleotide binding activity were the most enriched in the Molecular Function category (Figure 2). Biological Process annotations showed that biosynthesis, nucleobase-containing compound, transport, cellular protein modification process and response to stress were enriched in roots and leaves (Figure 2).

**Figure 3:**
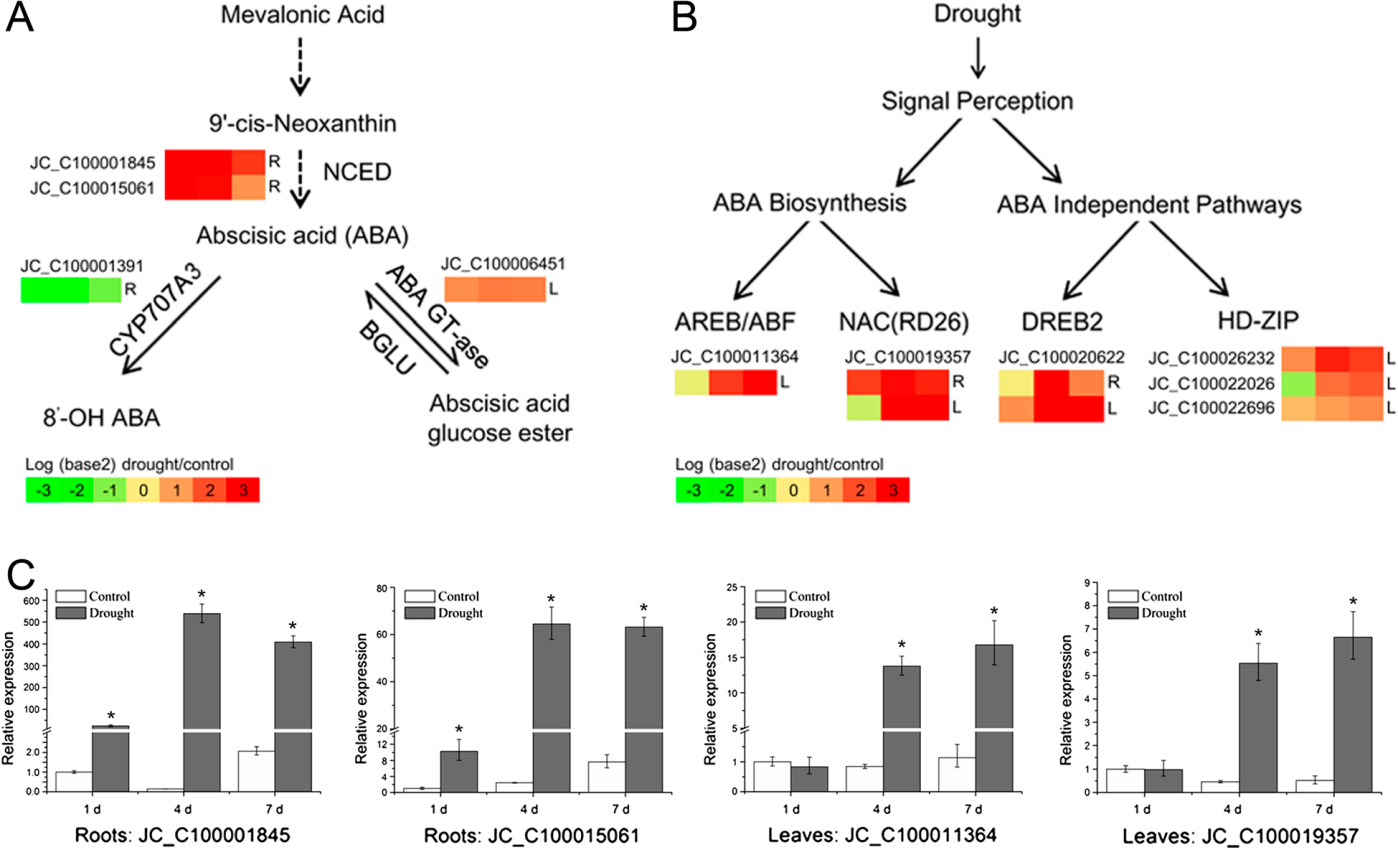
**ABA biosynthesis, catabolism and signal transduction and the results of quantitative real-time PCR (qRT-PCR). A.** ABA biosynthesis and catabolism; **B.** ABA signal transduction; **C.** qRT-PCR results for *NCEDs*, *ABF* and *RD26*. Relative expression level represents mean of n = 3 ± SD (Duncan test: *, P < 0.05). *NCED*, 9-cis-epoxycarotenoid dioxygenase; *BGLU*, beta glucosidase; *CYP707A3*, Cytochrome P450, family 707, subfamily A, polypeptide 3; *ABA GT-ase*, ABA Glycosyltransferase; *AREB*/*ABF*, abscisic acid responsive elements-binding factor; *RD26*, response to desiccation 26; *HD-ZIP*, homeodomain-leucine zipper protein; *DREB*, dehydration response element-binding protein.

To assess the accuracy of the digital expression data, genes involved in ABA biosynthesis and signal transduction (*NCEDs*, *AREB/ABF* and *RD26*) were tested by qRT-PCR (Figure 3). The results showed that two *NCED* genes (JC_C100001845 and JC_C100015061) were significantly up-regulated in roots under drought stress treatments, and two genes in an ABA dependent pathway, *ABF* (JC_C100011364) and *RD26* (JC_C100019357), were significantly up-regulated in leaves (Figure 3C). The gene expression patterns obtained from qRT-PCR confirmed the results of Digital Gene Expression Profiling, indicating that the gene expression profiling approach used in this study was a reliable method for analyzing the response of physic nut to drought stress.

### Drought stress responsive genes in roots

#### Abscisic acid (ABA) biosynthesis

Signal transduction is one of the GO terms most highly enriched during drought stress, and more than 50 DEGs with this annotation were identified (Figures 2, and 3, Additional file 3: Table S3). The roles of ABA in drought stress have been studied intensively. It has functions related to stomatal closure, osmotic adjustment and changes in metabolic pathways [3,9]. In the present study, the orthologs of *Arabidopsis NCED3* (JC_C100001845) and *NCED5* (JC_C100015061), which encode key enzymes in ABA biosynthesis, were observed to be strongly up-regulated in roots, whereas the ortholog of *Arabidopsis CYP707A3* (JC_C100001391), whose product catabolizes ABA, was down-regulated in roots (Figure 3, Additional file 3: Table S3). These results indicate that ABA was probably synthesized immediately upon the onset of drought stress and simultaneously its catabolism was suppressed in roots.

#### ABA signal transduction

In this study, two PP2C genes (JC_C100018292 and JC_C100005394), which are similar to AT1G07430 (clade A PP2C), were up-regulated in roots in all drought treatments (Additional file 3: Table S3). SnRK3 genes form one of the three clades of the SnRK family, which functions downstream of the PP2Cs and is probably involved in osmotic adjustment and ABA signaling [62,63]. Under drought stress in physic nut, three *ATSnRK3* orthologs (JC_C100025374, JC_C100006743, and JC_C100016558) were differentially expressed in roots (Additional file 3: Table S3). In addition, an ortholog (JC_C100019357) of *ANAC072*, which is also known as *RD26*, belonging to an ABA-dependent pathway, was up-regulated at 4 and 7 DAWI (Figure 3, Additional file 3: Table S3).

#### Transcription factors (TFs)

In addition to TFs associated with ABA-dependent pathways, one *DREB2C* (AT2G40340) ortholog (JC_C100020622) belonging to an ABA-independent pathway was up-regulated at 4 and 7 DAWI (Figure 3, Additional file 4: Table S4). There were also nine MYB family genes, seven basic helix-loop-helix (bHLH) family genes, six ethylene response factor (AP2/ERF) genes, six NAC family genes, and sixteen other TF genes that were differentially expressed in roots (Additional file 4: Table S4).

#### Osmotic adjustment

**Figure 4:**
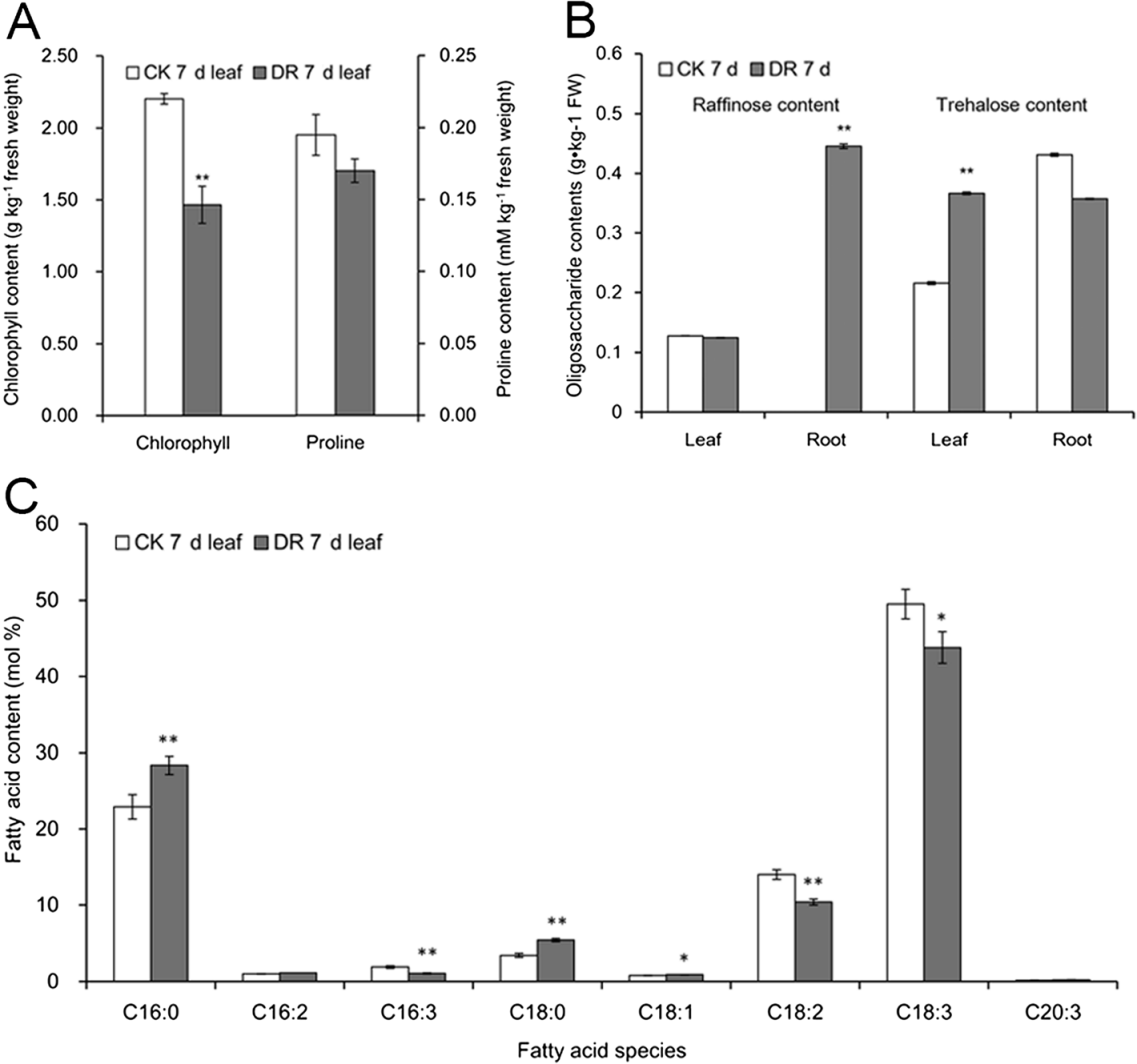
**Chlorophyll, proline, raffinose and trehalose contents and fatty acid composition of leaves 7 days after withholding irrigation. A.** Chlorophyll and proline contents of leaves 7 days after withholding irrigation; **B.** Raffinose and trehalose contents of leaves and roots 7 days after withholding irrigation determined by high performance liquid chromatography with evaporative light scattering detector (HPLC-ELSD); **C.** Major fatty acids (mol %) in leaves 7 days after withholding irrigation, detected by gas chromatography. Values represent mean of n = 3 ± SD (Duncan test: *, P < 0.05; **, P < 0.01). CK, control; DR, drought treatment; *FAT*, fatty acyl-ACP thioesterase; *SAD*, stearoyl-ACP desaturase; *FAD*, fatty acid desaturase; C16:0, palmitate; C16:2, hexadecadienoic acid; C16:3, hexadecatrienoic acid; C18:0, stearate; C18:1, oleate; C18:2, linoleic acid; C18:3, α-linolenic acid; C21:0, heneicosanoate; C20:3, homogamma linolenate.

Osmotic adjustment is crucial in plant resistance to drought stress and it contributes to water uptake and maintenance, membrane protection and ROS scavenging [64]. The genes related to osmotic adjustment that were up-regulated under drought stress in physic nut roots were mainly related to galactose and raffinose biosynthesis; they included JC_C100019062 (encoding galactinol synthase), JC_C100015469 and JC_C100008342 (encoding raffinose synthases) (Additional file 5: Table S5). Quantitative determination of oligosaccharides showed that the accumulation of raffinose was significantly increased in drought treated roots compared to control roots at 7 DAWI (Figure 4B).These data indicate that raffinose was important in conferring osmotic adjustment in the roots of physic nut seedlings under drought stress.

### Drought stress responsive genes in leaves

#### ABA signal transduction

In this study, two PP2C genes (JC_C100020478, JC_C100020994), which are similar to AT1G72770 and AT1G07430 (clade A PP2C), respectively, were up-regulated in leaves at 4 and 7 DAWI (Additional file 3: Table S3). Three SnRK3 genes (JC_C100025374, JC_C100006743, JC_C100016558) were up-regulated in leaves (Additional file 3: Table S3). In addition, two TF genes belonging to an ABA-dependent pathway, *ABF2/AREB1* (AT1G45249) ortholog JC_C100011364 and *ANAC072* ortholog JC_C100019357, and one ABA response gene, *RD22* ortholog JC_C100025279, were up-regulated at 4 and 7 DAWI (Figure 3, Additional file 3: Table S3).

#### Transcription factors

In leaves, as well as TFs belonging to ABA-dependent pathways, four TF genes belonging to ABA-independent pathways, *DREB2C* (AT2G40340) ortholog JC_C100020622, *ATHB1* orthologs JC_C100026232 and JC_C100022026, and *ATHB16* ortholog JC_C100022696, were up-regulated strongly at 4 and 7 DAWI (Figure 3, Additional file 4: Table S4). Additionally, there were thirteen zinc finger (ZF) genes, eight bHLH family genes, seven NAC family genes, five auxin response factor (ARF) genes, five GRAS family genes, and nineteen other TF genes that were differentially expressed in leaves, and most of them were up-regulated at 4 and 7 DAWI (Additional file 4: Table S4).

#### Osmotic adjustment

In leaves, one ortholog of Arabidopsis galactinol synthase *2* (JC_C100019062), two of raffinose synthase (JC_C100015469 and JC_C100008342), and one of stachyose synthase (JC_C100023767) were up-regulated in leaves (Additional file 5: Table S5). Three orthologs of trehalose synthase, JC_C100026838, JC_C100025852 and JC_C100026150, were up-regulated at 7 DAWI (Additional file 5: Table S5). As a result of quantitative determination of oligosaccharides, we observed an increased accumulation of trehalose, but not raffinose, in drought treated leaves compared to control leaves at 7 DAWI (Figure 4B). The content of stachyose was very low in the tissues tested. The lack of an increase in accumulation of raffinose and stachyose may be because they are transported out of leaves for use as carbohydrate sources in other tissues; alternatively their biosynthesis may be regulated at the post-transcriptional level. In addition, a glutamate decarboxylase (AT3G17760) ortholog (JC_C100001997), whose product is involved in 4-aminobutanoate biosynthesis, was up-regulated in leaves at 4 and 7 DAWI (Additional file 5: Table S5). Genes related to proline biosynthesis were not differentially expressed, except that JC_C100022037, the ortholog of *ATP5CDH* (Delta-pyrroline-5-carboxylate dehydrogenase) which is involved in the catabolism of proline, was up-regulated during the drought treatments (Additional file 5: Table S5). When we measured the proline content in leaves of 7 DAWI seedlings, we observed no significant difference between control and drought stress seedlings (Figure 4A).

#### Endoplasmic reticulum (ER) stress responses

The ER stress response is activated by unfolded proteins that accumulate in the ER when plants are exposed to adverse environments [24]. There are two signal transduction pathways mediating this response in plants, and in *Arabidopsis* the genes involved are *bZIP17*, *bZIP28* and *IRE1*, *bZIP60*, respectively [24-26]. The genes involved in the mobilization of bZIP28 are *BiP* (Binding Protein, which releases bZIP28 when misfolded proteins are accumulated), *SAR* (Sar GTPase), genes encoding COPII vesicle elements, *S1P* (site 1 protease) and *S2P* [24]. bZIP28 up-regulates genes that encode components of the ER protein-folding machinery, including *BIP3*, *CRT* (Calreticulin), *PDI* (Protein Disulfide Isomerase) and genes encoding CCACG and CCAAT box binding factors, such as NF-YA, NF-YB, and NF-YC [24]. In this study, the orthologs of *bZIP17* (JC_C100001008), *IRE1* (JC_C100006072) and *bZIP60* (JC_C100010582) were significantly up-regulated in leaves at 4 and 7 DAWI (Additional file 6: Table S6). The orthologs of genes involved in the mobilization of bZIP28, *NF-YB3* (JC_C100019844 and JC_C100019844), *BiP* (JC_C100018784), *SAR1* (JC_C100010380), *PDI1* (JC_C100003154), *PDI8* (JC_C100002910), *PDI11* (JC_C100024282), and *CRT3* (JC_C100004920) were significantly up-regulated at 7 DAWI (Additional file 6: Table S6). Orthologs of genes related to autophagy, *BAG* (JC_C100023607) and *ATG* (JC_C100007965, JC_C100008273, JC_C100019709, JC_C100018832 and JC_C100009284), were up-regulated in drought stress, especially at 4 and 7 DAWI (Additional file 6: Table S6). In addition, about 10 genes involved in ER stress responses were differentially expressed in roots under drought treatments (Additional file 6: Table S6).

#### Ethylene (ETH) biosynthesis and signal transduction

ETH plays important roles in regulating plant development and metabolism, including leaf senescence, leaf abscission, and secondary metabolism [18,65]. In plants, ETH is synthesized from L-methionine by a number of enzymes, including 1-Aminocyclopropane-1-Carboxylate Synthase (ACS) and Aminocyclopropane Carboxylate Oxidase (ACO) [19,20]. Its functions are mediated through ETH receptors (ethylene response proteins, ETRs, ethylene response sensors, ERSs, and ethylene insensitive 4, *EIN4*) [23] and Ethylene-Responsive Transcription Factors (ERFs) [21,22,66]. In this study, three ACO orthologs (JC_C100008985, JC_C100009042, and JC_C100026451), two ETR orthologs (JC_C100002132 and JC_C100014744), one ERS ortholog (JC_C100024264), four EIN orthologs (JC_C100018907, JC_C100012212, JC_C100011749 and JC_C100000778) and one ERF ortholog (JC_C100026088) were significantly up-regulated in leaves at 7 DAWI (Additional file 7: Table S7).

#### Chlorophyll degradation

We found that the orthologs of *SGR* (stay green) (JC_C100017301), *NYC1* (non-yellow coloring 1) (JC_C100024913), *PPH* (pheophytinase) (JC_C100001552), *PAO* (pheide a oxygenase) (JC_C100021098), and *RCCR* (red chlorophyll catabolite reductase) (JC_C100017722) were up-regulated at 4 and 7 DAWI (Additional file 7: Table S7). These genes encode major components of the chlorophyll degradation pathway in plants. We therefore measured the chlorophyll content of leaves from 7 DAWI seedlings. The results showed that the total chlorophyll content in drought treated seedlings was significantly lower than that in control seedlings (Figure 4A).

#### Photosynthesis, glycolysis and tricarboxylic acid (TCA) cycle

Photosynthesis, glycolysis and the TCA cycle are the basic physiological processes that provide ATP and intermediates for plant metabolism. In this study, genes encoding photosystem I, photosystem II and Calvin cycle components were found to be significantly down-regulated in leaves at 7 DAWI; they included eleven genes putatively related to LHC (light-harvesting complex) proteins, and genes encoding key enzymes in the Calvin cycle, *RBCS* (ribulose bisphosphate carboxylase small chain) (JC_C100010075 and JC_C100007353), *PGK* (phosphoglycerate kinase) (JC_C100005831) and *PRK* (phosphoribulokinase) (JC_C100003780) (Additional file 7: Table S7). With respect to glycolysis and the TCA cycle, several genes were up-regulated at 4 and 7 DAWI, including *PFK* (6-phosphofructokinase) (JC_C100025529, JC_C100016388), *ACO* (aconitate hydratase) (JC_C1000 19761), and *DLST* (dihydrolipoamide succinyltransferase) (JC_C1000 02574) (Additional file 7: Table S7).

#### Wax biosynthesis

An increase in leaf wax content has been observed in many plants under drought stress [28,30,67]. In this study, a number of genes related to wax biosynthesis, wax transport and the regulation of these processes were up-regulated, especially at 4 and 7 DAWI, including a *MYB96* ortholog (JC_C100021705), a *CER* ortholog (JC_C100022748, JC_C100009202, and JC_C100024323), a *MAH* (cytochrome P450, family 96, subfamily A) ortholog (JC_C100014199), a *WSD* (Wax-ester synthase) ortholog (JC_C100003255) and an *ABCG* (Arabidopsis thaliana white-brown complex homolog protein) ortholog (JC_C100006655) (Additional file 8: Figure S1, Additional file 9: Table S8).

#### Fatty acid composition

Plant fatty acid composition has been reported as being changed under drought stress, with examples such as the increase in saturated fatty acid content and the decrease in amount of unsaturated fatty acid observed in aerial parts of *Carthamus tinctorius* [68] and in *Salvia officinalis* [69]. However, the opposite trend was reported in Kentucky Bluegrass [32]. In the present study, genes related to polyunsaturated fatty acid synthesis were found to be down-regulated in leaves under drought stress; they included *FAD2* (fatty acid desaturase 2) (JC_C100004186), *FAD4* (JC_C100019742), *FAD5* (JC_C100013404), *FAD6* (JC_C100009152), *FAD8* (JC_C100009540), and *FATA* (JC_C100022895) (Additional file 9: Table S8). We therefore analyzed the fatty acid composition of leaves at 7 DAWI. The results showed that the proportion of polyunsaturated fatty acids (C16:3, C18:2, C18:3) in drought treated leaves was significantly lower than that in control leaves, while saturated fatty acids (C16:0, C18:0) showed the opposite pattern (Figure 4C).

## Discussion

Gene expression profiling provides a large amount of transcriptional information, and makes it possible to look into the complex networks of gene regulation that operate under different environments [9]. For instance, 1240 ESTs generated from root cDNA libraries prepared from physic nut showed that the majority of TFs had sequence similarity to genes known to be involved in abiotic and biotic stress in other plant species [70]. To further shed light on the molecular mechanism by which physic nut seedlings respond to drought stress, gene expression profiles of a total of 24 samples (two biological replicates of each of 12 samples) were constructed. After analyzing the DEGs systematically, we inferred that ABA and ETH synthesis and signal transduction, raffinose and trehalose synthesis, leaf senescence and abscission, ER stress responses and lipid metabolism play important roles in physic nut seedlings under drought stress.

Plant hormones and other signal molecules are important in drought responses in plants [6,71]. The most important hormone involved in these responses is ABA [9,72]. In *Arabidopsis*, when plants suffered drought stress, the expression of *ATZEP* and *ATNCED3* was up-regulated significantly and the production of ABA in roots was increased [16]. ABA induces the expression of many TF genes, including those encoding proteins of the MYB, MYC, NAC, and ABF/AREB families. These TFs then induce the expression of downstream genes, such as *RD22*, *RD29B*, and *RD20A* [6,71]. In our experiments, some genes involved in ABA biosynthesis, the *NCED*s, were strongly up-regulated in roots, and genes involved in ABA signal transduction, *PP2C*, *SnRK3*, *ABF*, *RD22* and *RD26*, were up-regulated in both roots and leaves during the drought treatments (Figure 3, Additional file 3: Table S3). These results indicate that ABA plays a crucial role in the process of response to drought stress in physic nut seedlings. In addition, there are ABA-independent drought response pathways in plants, involving genes that include the *HD-ZIP* family, *DREB2* and some *NAC* TF genes [6]. In drought stress in physic nut seedlings, a *DREB2* gene was up-regulated in both roots and leaves, and four *HD-ZIP* genes were up-regulated in leaves, indicating that components of ABA-independent pathways participate in the process of drought adaptation (Figure 3B). The similarity of the regulation of ABA signal transduction between *Arabidopsis* and physic nut indicated that these signaling pathways are conserved across the two species.

Among the processes taking place downstream of this transcriptional regulatory network, large amounts of osmoprotectants are synthesized which, depending on plant species, can include proline (Pro), γ-aminobutyric acid (GABA), glycine betaine (GB), trehalose (Tre) and raffinose family oligosaccharides (RFO) [3]. Previous studies showed that total soluble sugar content increased dramatically during drought stress in physic nut, and it has been regarded as the primary osmoprotectant underlying drought resistance in this species, whereas Pro, GB and other amino acids are not particularly important [38]. However, Wang et al. [73] found that proline was synthesized to, and maintained at, high levels to mitigate the damage caused by drought stress when physic nut seedlings from low-nitrogen conditions were suddenly exposed to PEG-6000. Our data indicated that genes involved in proline synthesis were not significantly up-regulated in roots or leaves (Additional file 5: Table S5), and the proline content showed no significant difference between control and treated leaves at 7 DAWI (Figure 4A). These results imply that proline may play little part in osmotic adjustment in physic nut plants under drought stress, and that the findings of Wang et al. [73] may be due to the nature of the stress imposed by PEG-6000. Genes involved in the biosynthesis of trehalose and raffinose were significantly up-regulated in roots and leaves (Additional file 5: Table S5), indicated that these compounds may have major impacts on osmotic adjustment and ROS scavenging [56] during drought stress in physic nut seedlings.

ER stress triggers the unfolded protein response (UPR), which can both reduce the load of unfolded protein in the ER by enhancing protein folding and minimize the damage caused by unfolded proteins by inducing cell autophagy [24,74]. So far, the exact functions of UPRs in drought stress have not been identified, but they are thought to mitigate the damage caused by stress [24]. For instance, over-expression of the gene encoding ER luminal binding protein (BiP) increased the drought tolerance of soybean [75], and autophagy is considered to be a key process in drought tolerance in *Arabidopsis* [76]. In this study, the key genes involved in ER stress, *BiP1*, *bZIP60*, *bZIP28*/*bZIP17*, and downstream autophagy-related genes, were up-regulated at 4 and 7 DAWI, especially in leaves (Additional file 6: Table S6). These changes at the transcriptional level indicated that UPRs are up-regulated in physic nut seedlings, where they can efficiently degrade unfolded proteins and keep organelles working normally. As drought stress continues, UPR is probably associated with cell death in leaves and the process of leaf-drop.

ETH has functions in plant growth [17], leaf senescence [77,78] and leaf abscission [79]. Physic nut plants show drought avoidance behavior, including a strategy of water saving through strict stomatal regulation and drought-induced leaf drop [43,44]. The up-regulation of genes related to ethylene biosynthesis, signaling and ethylene response transcription factor genes suggests that they are probably involved in leaf senescence and abscission in physic nut during drought stress (Additional file 7: Table S7). Genes related to chlorophyll breakdown were up-regulated at 4 and 7 DAWI (Additional file 7: Table S7), and the leaf chlorophyll content was significantly decreased by 7 DAWI (Figure 4A). These results indicate that drought stress induced leaf senescence. Furthermore, genes related to photoreactions and the Calvin cycle were significantly down-regulated, whereas genes related to glycolysis and the TCA cycle were up-regulated in leaves, especially at 7 DAWI (Additional file 7: Table S7). This phenomenon also indicated that the leaves began to senesce under drought stress, and this was especially marked at 7 DAWI [80]. These data suggest that ETH may play important roles in drought avoidance in physic nut plants under serious drought stress, which results in leaf senescence and leaf drop.

Wax is the outermost thin hydrophobic layer that protects leaves from nonstomatal water loss during drought [29,30]. It is synthesized in *Arabidopsis* by a pathway terminating in the enzyme encoded by *WSD* and its synthesis is regulated by *MYB96* and *CER* (Additional file 8: Figure S1), especially under drought stress [27,28]. In our study, we found dozens of DEGs involved in wax biosynthesis (Additional file 8: Figure S1, Additional file 9: Table S8,). At 4 and 7 DAWI, genes involved in wax biosynthesis (*KCS*, *WSD*) and its regulation (*MYB96, CER*) were up-regulated more than 4-fold in leaves (Additional file 9: Table S8). This result showed that physic nut seedlings have a similar regulatory mechanism for wax synthesis to that of *Arabidopsis* when plants are exposed to drought stress. The synthesis of wax would strengthen the hydrophobic barrier that prevents non-stomatal water loss and increase plant drought tolerance [81,82].

Fatty acid metabolism may be involved in plant adaption to drought stress, and increased unsaturated fatty acid contents are believed to to maintain the fluidity and stability of cellular membranes during plant adaptation to drought stress [31,32]. Previous studies showed that 1 μM L^−1^ ABA treatment reduced the transcription of *FAD2* [83], and that overexpression of *FAD2* and *FAD3* in *Arabidopsis* increased the proportion of linoleic acid (C18:2) and α-linolenic acid (C18:3), respectively, in seeds [60]. In this study, genes encoding fatty acid desaturase (FAD), such as *FAD2*, *FAD4*, *FAD5*, *FAD6* and *FAD8*, were down-regulated at 4 and 7 DAWI (Additional file 9: Table S8). The down-regulation of genes related to polyunsaturated fatty acid biosynthesis may be a result of ABA signaling, and it is consistent with the observed decreases in polyunsaturated fatty acid content (Figure 4C, Additional file 9: Table S8).

In the present study, we also observed that the responses of genes in many pathways differed between leaves and roots under drought stress. The orthologs of *Arabidopsis NCED3* (JC_C100001845) and *NCED5* (JC_C100015061) were up-regulated significantly in roots, but not in leaves (Additional file 3: Table S3) under drought. This result indicated that ABA was synthesized mainly in roots under drought stress treatments. At 7 DAWI, raffinose was accumulated in roots while trehalose was accumulated in leaves (Figure 4B). In addition, numerous genes involved in ETH synthesis and signal transduction, chlorophyll degradation, glycolysis, tricarboxylic acid cycle, and wax synthesis were up-regulated only in leaves, and genes involved in photosynthesis, Calvin cycle, fatty acid synthesis were down-regulated only in leaves (Additional file 6: Table S6, Additional file 9: S8). The differences between roots and leaves indicated tissue specificities in drought responses, which may contribute to the drought tolerance of physic nut seedlings.

On the other hand, we also found that ABA biosynthetic genes were up-regulated from 1 DAWI (Additional file 3: Table S3, Additional file 5: S5), whereas genes related to ETH signal transduction, energy metabolism, osmoprotectant biosynthesis, wax synthesis, chlorophyll degradation, etc., showed changes in expression mainly at 4 and 7 DAWI in leaves (Additional file 3: Table S3, Additional file 6: S6, Additional file 9: S8). These results indicated that in physic nut the biosynthesis of ABA is a rapid response to drought stress. As drought stress became more severe, genes with products involved in energy metabolism, osmotic balance, changes in cell structure, and leaf senescence and abscission showed altered expression in leaves.

## Conclusion

**Figure 5:**
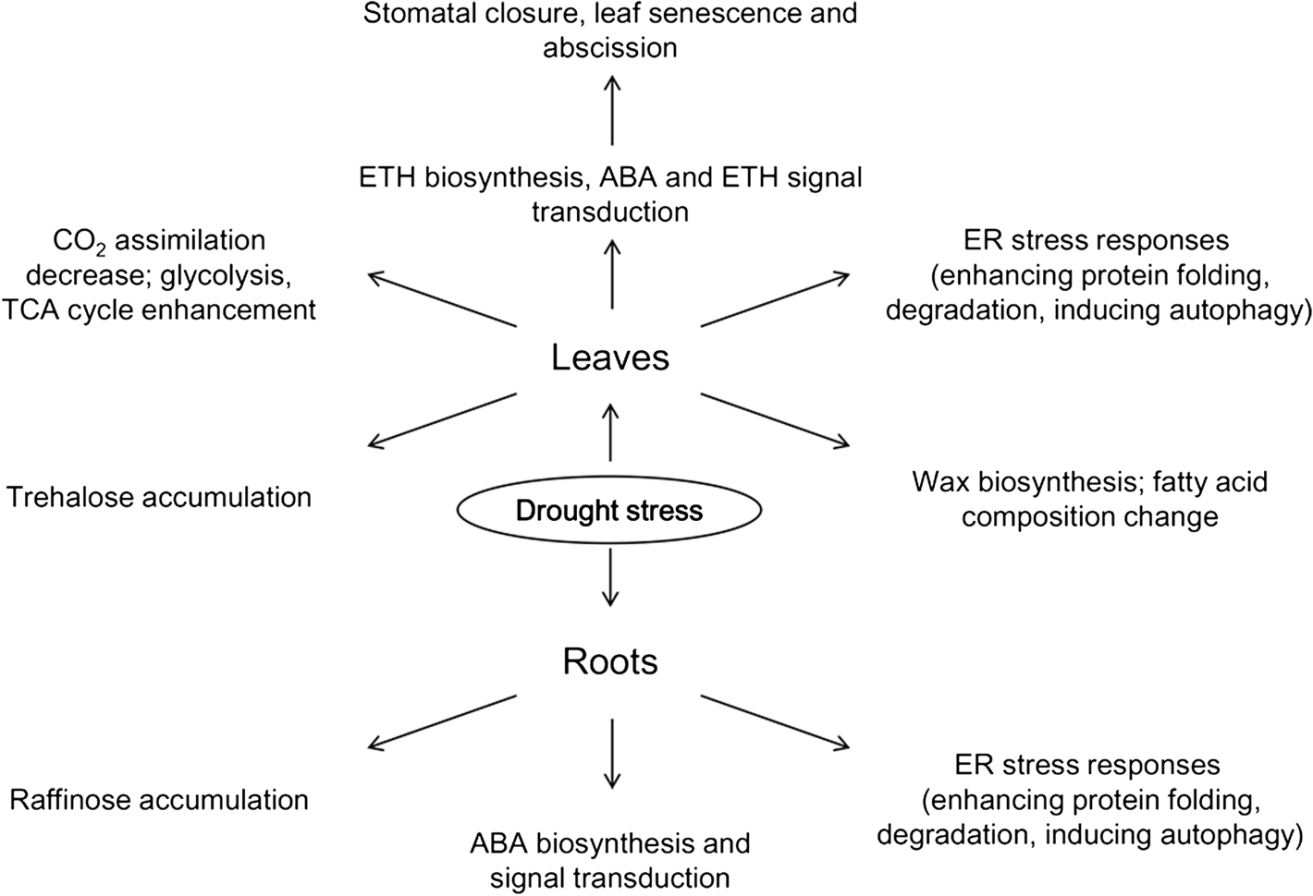
**Transcriptional and physiological pathways leading to drought resistance in** ***J. curcas.*** In roots, ABA synthesis and signal transduction was enhanced, and raffinose synthesis was significantly up-regulated. In leaves, ETH was synthesized, and ABA and ETH signal transduction pathways were enhanced. The ER stress response was activated, probably to remove unfolded proteins or to induce autophagy. Trehalose was accumulated, and glycolysis and the TCA cycle were enhanced, whereas chlorophyll content and CO_2_ assimilation decreased. The proportion of polyunsaturated fatty acids decreased and wax synthesis increased. ABA, abscisic acid; ETH, ethylene; TCA Cycle, tricarboxylic acid cycle; ER, endoplasmic reticulum.

In total, 4103 differentially expressed genes were identified in the transcriptomic profiles which we constructed by RNA sequencing. The analysis of specific pathways, combined with physiological data, allowed us to identify the transcriptional and physiological pathways leading to drought resistance in physic nut seedlings. In roots, ABA synthesis and signal transduction was enhanced, and raffinose accumulated significantly. In leaves, ETH was synthesized, and ABA and ETH signal transduction pathways were enhanced. The ER stress response was activated, probably to remove unfolded proteins or to induce autophagy. Trehalose was accumulated, and glycolysis and the TCA cycle were enhanced, whereas chlorophyll content and CO_2_ assimilation decreased. The proportion of polyunsaturated fatty acids decreased and wax synthesis increased (Figure 5). These results have provided us with much information as a basis for understanding the mechanisms of drought resistance in physic nut, and also provided us with new tools with which to improve the drought resistance of physic nut and other plants.

## Additional files

Additional file 1: Table S1. Sequencing saturation analysis. Additional file 2: Table S2. Genes differentially expressed in more than one tissue and/or drought condition. Additional file 3: Table S3. List of DEGs involved in abscisic acid biosynthesis and signal transduction. Additional file 4: Table S4. List of DEGs involved in transcriptional regulation. Additional file 5: Table S5. List of DEGs involved in osmotic adjustment. Additional file 6: Table S6. List of DEGs involved in endoplasmic reticulum stress. Additional file 7: Table S7. List of DEGs involved in ethylene synthesis and response, chlorophyll degradation, Calvin cycle, glycolysis and tricarboxylic acid cycle. Additional file 8: Figure S1. Fatty acid elongation and wax biosynthesis. A. Pathway of fatty acid elongation and wax biosynthesis; B. Differentially expressed genes related to fatty acid elongation and wax biosynthesis. *FAS*, fatty acid synthesis; *LACS*, long-chain acyl-CoA synthetase; ER, endoplasmic reticulum; *ACC*, acetyl-CoA carboxylase; *ECR*, enoyl-CoA reductase; *HACD*, hydroxyacyl-CoA dehydratase; *KCR*, ketoacyl-CoA reductase; *KCS*, ketoacyl-CoA synthase; *WSD*, wax-ester synthase / diacylglycerol O-acyltransferase; *MAH*, cytochrome P450, family 96, subfamily A; *ABCG*, *Arabidopsis thaliana* white-brown complex homolog protein; *WIN1*/*SHN1*, SHN Transcription Factor 1; *CER*, eceriferum. Additional file 9: Table S8. List of DEGs involved in fatty acid synthesis, elongation and desaturation and in wax biosynthesis.

## Abbreviations

DEG: differentially expressed gene
TPM: number of transcripts per million tags
GO: Gene Ontology
qRT-PCR: quantitative real-time PCR
HPLC-ELSD: high performance liquid chromatography with evaporative light scattering detector
ABA: abscisic acid
ER: endoplasmic reticulum
ETH: ethylene
ROS: reactive oxygen species
TF: transcription factor
DAWI: days after withholding irrigation
