# Supplementary material for: Global analysis of gene expression profiles in physic nut (Jatropha curcas L.) seedlings exposed to drought stress
Source: BMC Plant Biol. 2015 Jan 21;15:17. doi: 10.1186/s12870-014-0397-x (PMC4307156; doi:10.1186/s12870-014-0397-x)
Supplement: Additional file 1: Table S1. — Sequencing saturation analysis. [file 12870_2014_397_MOESM1_ESM.pdf]

Table S1. Sequencing saturation analysis

Tissue	Control	Drought stress
Root - 1 d - replication 1	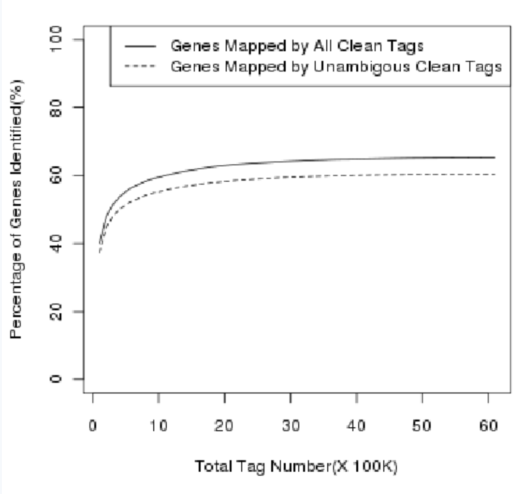	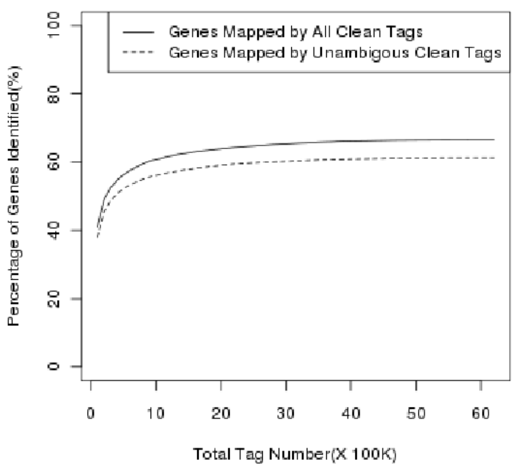
Root - 1 d - replication 2	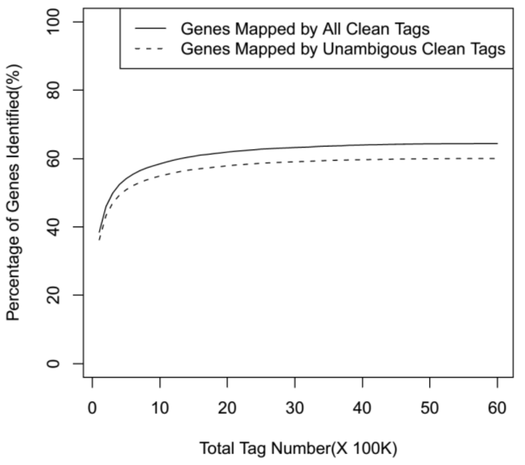	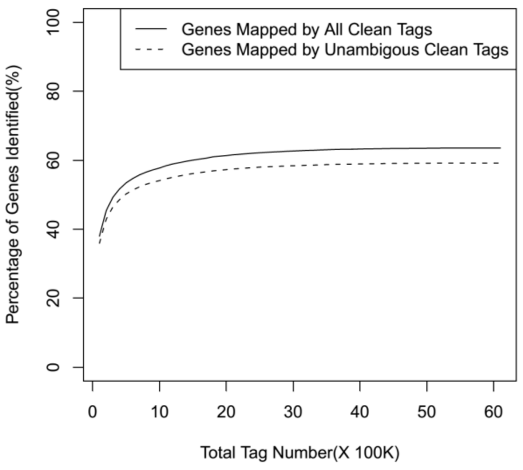
Root - 4 d - replication 1	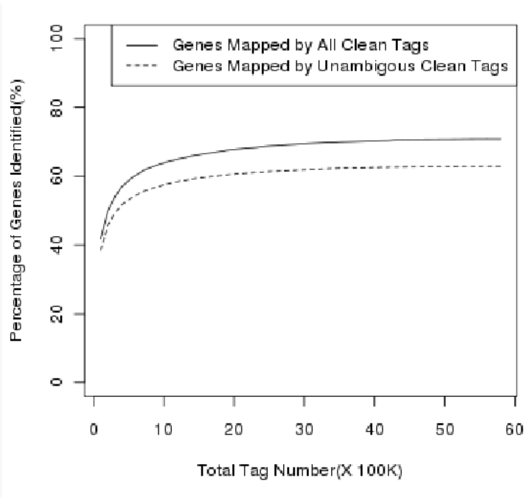	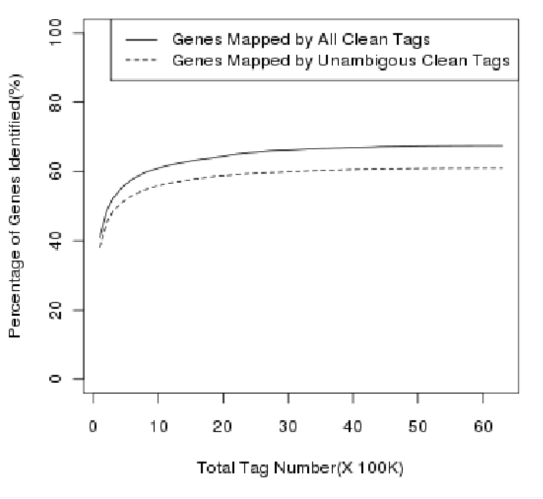

<table>
<tr><td>Root - 4 d - replication 2</td><td>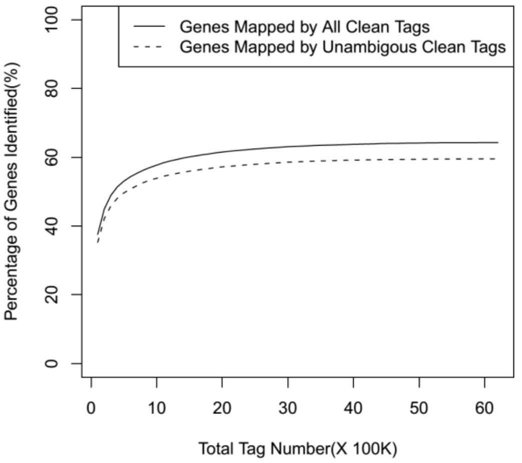
</td><td>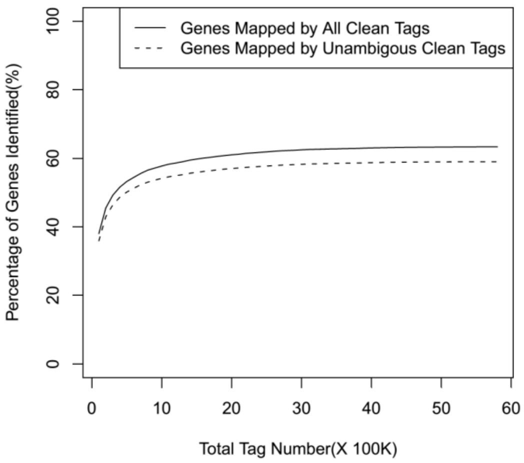
</td></tr>
<tr><td>Root - 7 d - replication 1</td><td>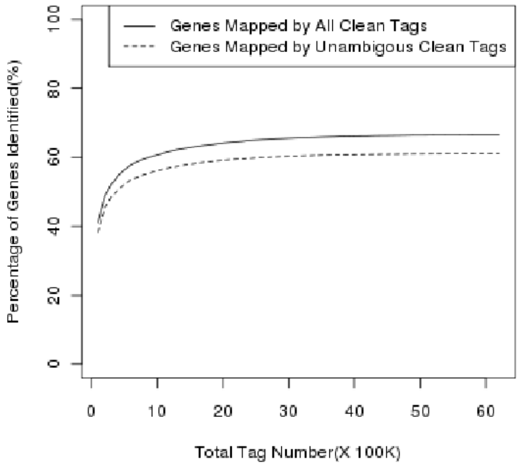
</td><td>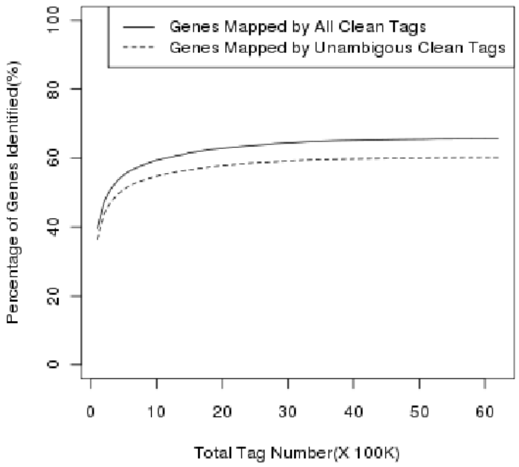
</td></tr>
<tr><td>Root - 7 d - replication 2</td><td>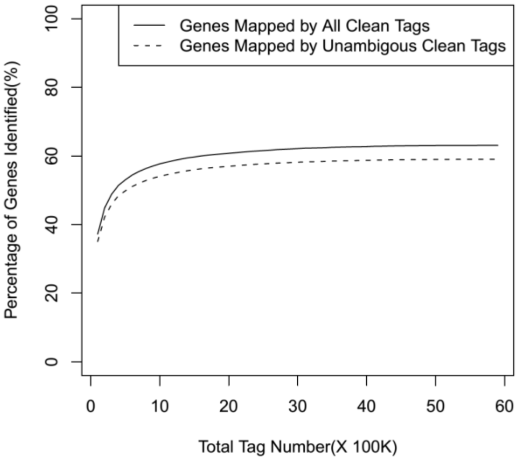
</td><td>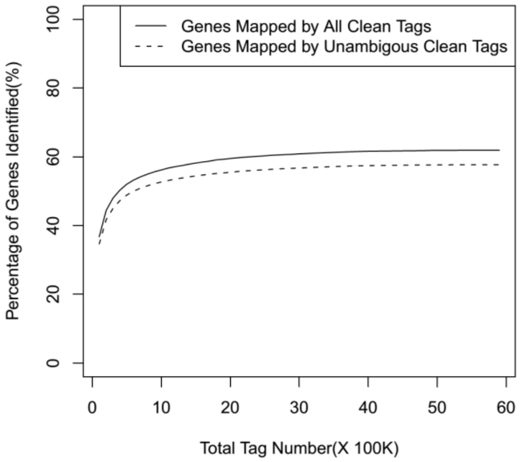
</td></tr>
<tr><td></td><td></td><td></td></tr>
</table>

Leaf - 1 d - replication 1	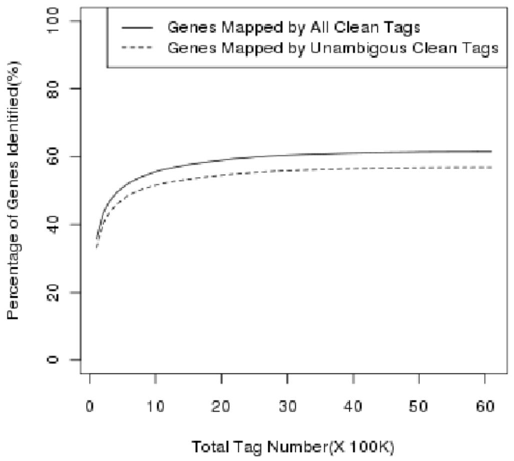	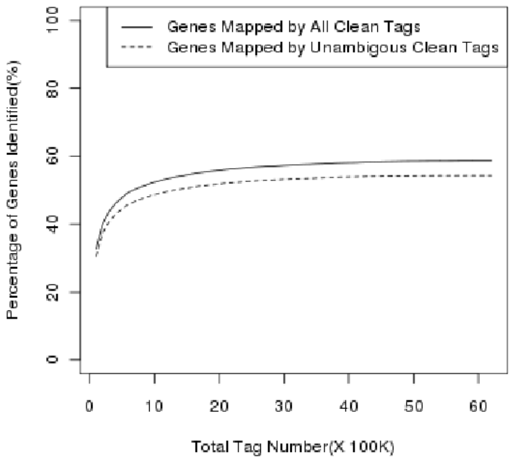
Leaf - 1 d - replication 2	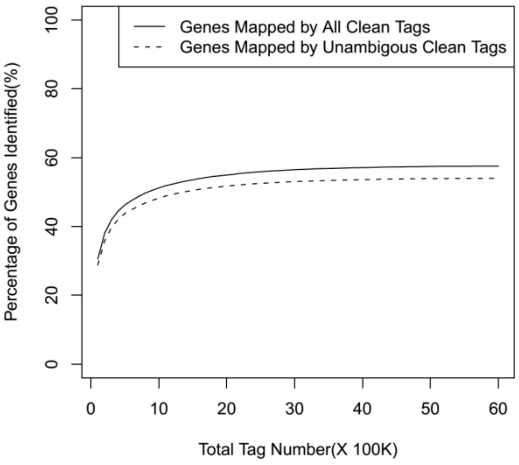	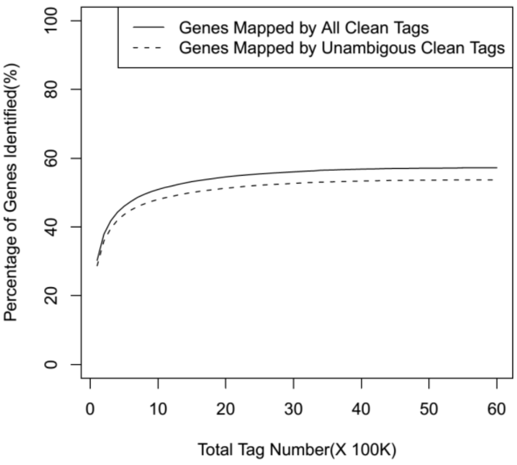
Leaf - 4 d - replication 1	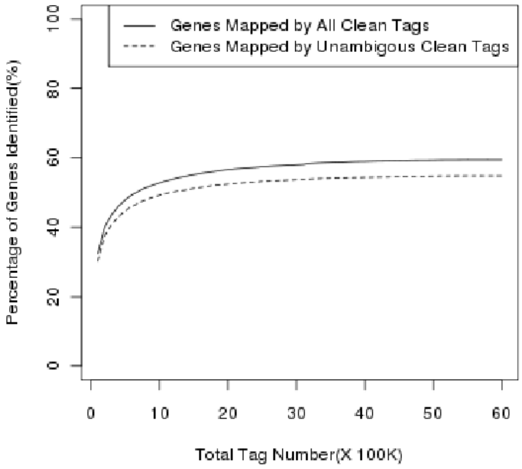	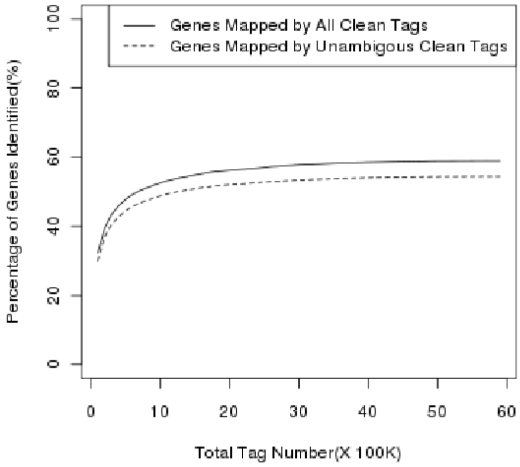
Leaf - 4 d - replication 2		

<table>
<tr>
<td></td>
<td>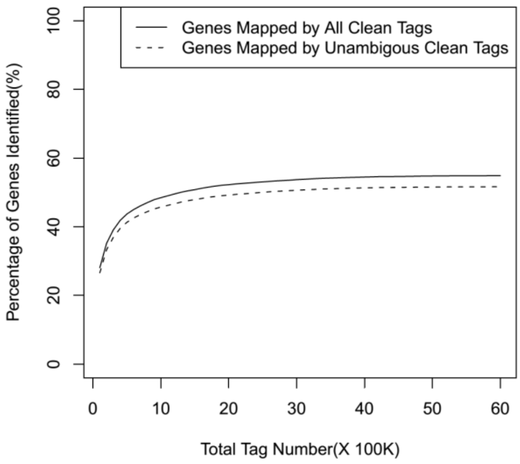
</td>
<td>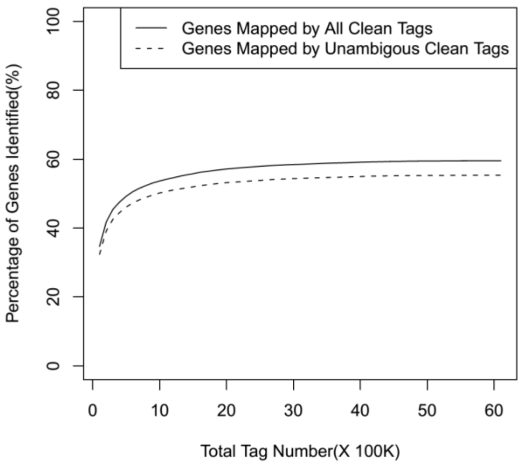
</td>
</tr>
<tr>
<td>Leaf - 7 d - replication 1</td>
<td>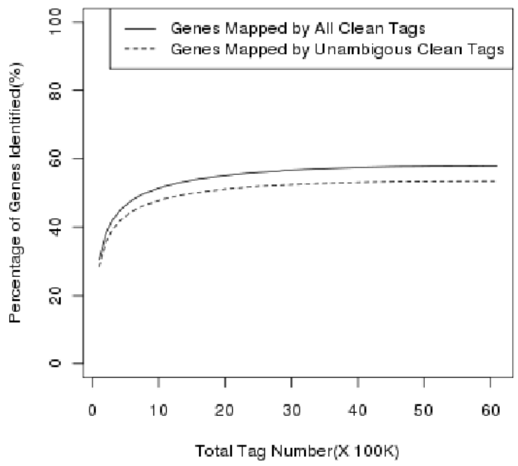
</td>
<td>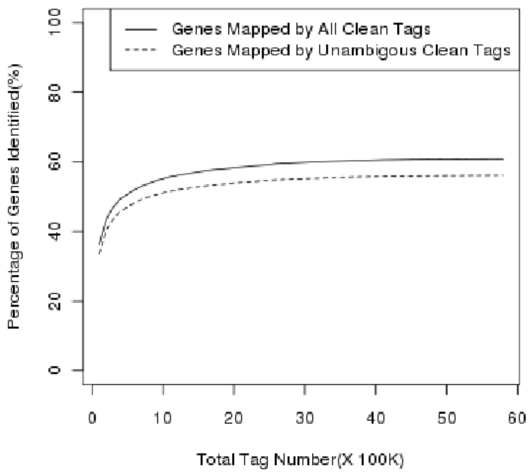
</td>
</tr>
<tr>
<td>Leaf - 7 d - replication 2</td>
<td>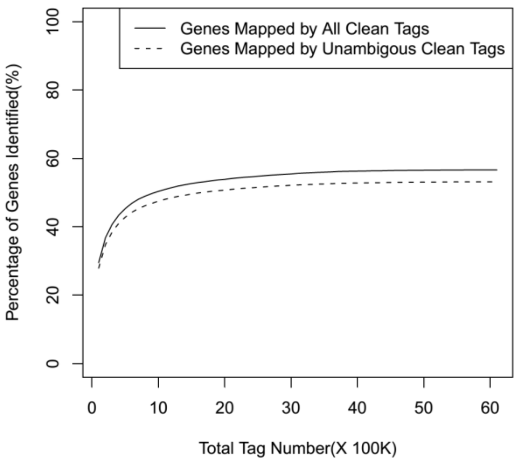
</td>
<td>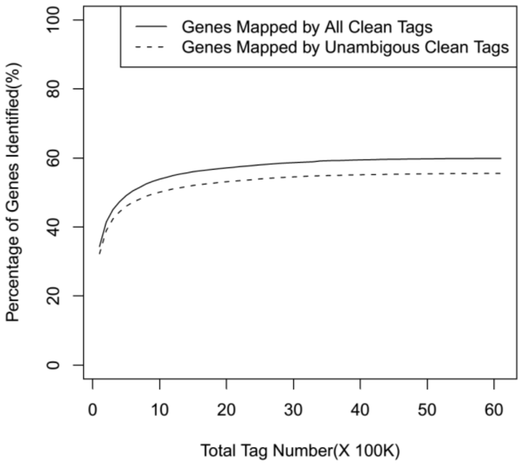
</td>
</tr>
</table>
