# Supplementary figures and images for: Global analysis of gene expression profiles in physic nut (Jatropha curcas L.) seedlings exposed to drought stress
Source: BMC Plant Biol. 2015 Jan 21;15:17. doi: 10.1186/s12870-014-0397-x (PMC4307156; doi:10.1186/s12870-014-0397-x)

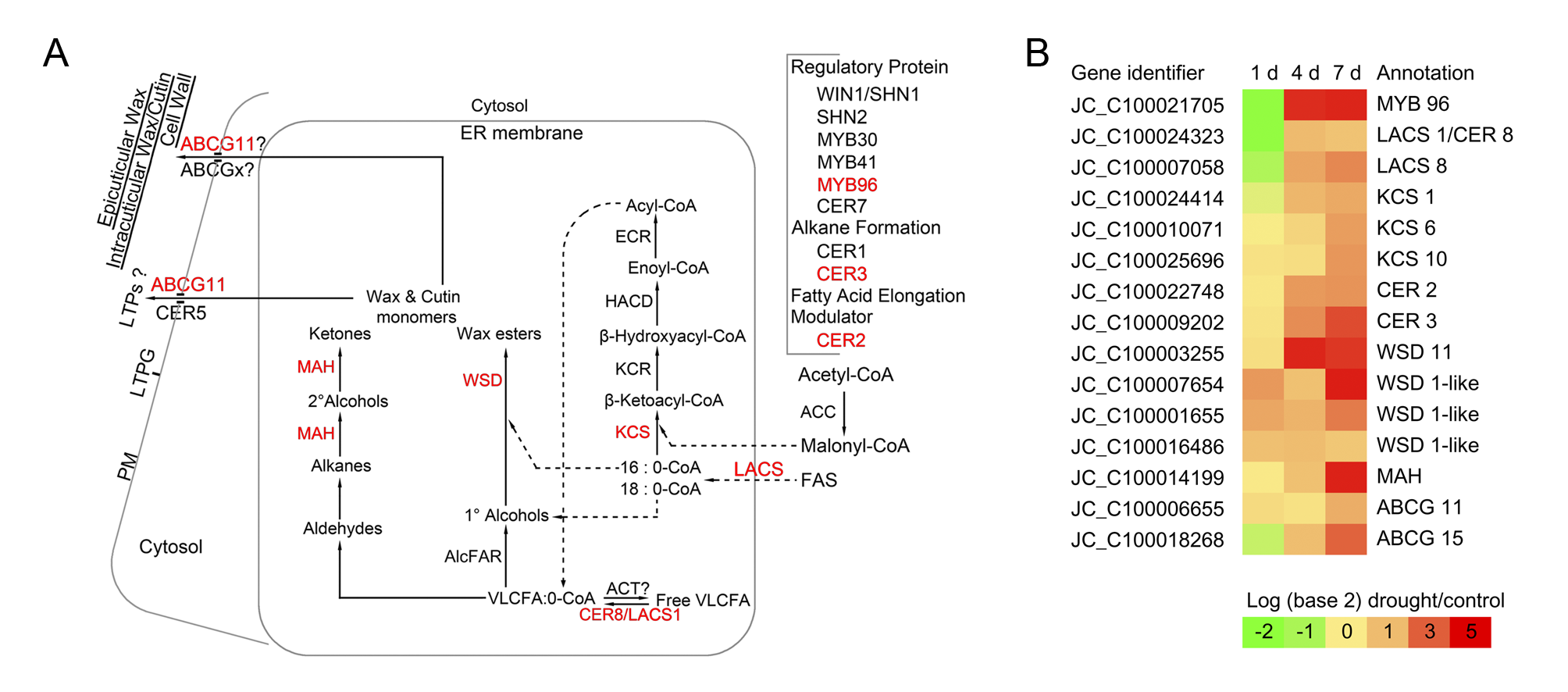

Supplement: Additional file 8: Figure S1. — Fatty acid elongation and wax biosynthesis. A. Pathway of fatty acid elongation and wax biosynthesis; B. Differentially expressed genes related to fatty acid elongation and wax biosynthesis. FAS, fatty acid synthesis; LACS, long-chain acyl-CoA synthetase; ER, endoplasmic reticulum; ACC, acetyl-CoA carboxylase; ECR, enoyl-CoA reductase; HACD, hydroxyacyl-CoA dehydratase; KCR, ketoacyl-CoA reductase; KCS, ketoacyl-CoA synthase; WSD, wax-ester synthase / diacylglycerol O-acyltransferase; MAH, cytochrome P450, family 96, subfamily A; ABCG, Arabidopsis thaliana white-brown complex homolog protein; WIN1/SHN1, SHN Transcription Factor 1; CER, eceriferum. [file 12870_2014_397_MOESM8_ESM.tiff]
